# TIMM44 is a potential therapeutic target of human glioma

**DOI:** 10.7150/thno.78616

**Published:** 2022-10-31

**Authors:** Yi-zhuo Guo, Gang Chen, Man Huang, Yin Wang, Yuan-yuan Liu, Qin Jiang, Cong Cao, Fang Liu

**Affiliations:** 1 Institute of Neuroscience, Soochow University, Suzhou, and Department of Neurosurgery, The affiliated Changzhou No. 2 People's Hospital of Nanjing Medical University, Changzhou, China.; 2 Department of Neurosurgery, the First Affiliated Hospital of Soochow University, Suzhou, China.; 3 Department of Oncology, Dushu Lake Hospital Affiliated of Soochow University, Suzhou, China.; 4 Department of Radiotherapy and Oncology, Affiliated Kunshan Hospital of Jiangsu University, Kunshan, China.; 5 The Fourth School of Clinical Medicine, The Affiliated Eye Hospital, Nanjing Medical University, Nanjing, China.; 6 Jiangsu Key Laboratory of Neuropsychiatric Diseases, Clinical Research Center of Neurological Disease, The Second Affiliated Hospital of Soochow University, Suzhou, China.

**Keywords:** Glioma, TIMM44, Mitochondria, Therapeutic target

## Abstract

TIMM44 (translocase of inner mitochondrial membrane 44) is essential for the maintenance of mitochondrial functions. Bioinformatics studies and results from the local high-grade glioma tissues showed that *TIMM44* mRNA and protein levels are elevated in glioma, correlating with poor overall survival. Mitochondrial TIMM44 upregulation was also detected in patient-derived primary glioma cells and immortalized cell line. In primary and established glioma cells, TIMM44 depletion, using the lentiviral shRNA strategy or the CRISPR/Cas9 knockout (KO) method, robustly inhibited cell viability, proliferation and migration. Moreover, TIMM44 silencing/KO resulted in mitochondrial complex I inhibition, ATP depletion, mitochondrial membrane potential reduction, oxidative stress and DNA damage, and eventually provoked apoptosis. Conversely, ectopic overexpression of TIMM44 augmented glioma cell proliferation and migration. TIMM44 upregulation in glioma is possibly due to increased TIMM44 transcriptional machinery by the transcription factor GATA3 in a YME1L (YME1 Like 1 ATPase)-dependent manner. *In vivo*, the growth of subcutaneous glioma xenografts was suppressed after intratumoral injection of TIMM44 shRNA adeno-associated virus (AAV). TIMM44 depletion, ATP reduction, oxidative injury and apoptosis were detected in TIMM44 shRNA AAV-injected glioma xenografts. Moreover, the intracranial growth of TIMM44 KO glioma cells in the mouse brain was largely inhibited. Together, overexpressed TIMM44 could be a novel and promising therapeutic target of human glioma.

## Introduction

In central nerve system (CNS), glioma with astrocytic or oligodendroglial origin is the most common malignancy 1, 2. The current clinical treatments for glioma include tumor resection, radiation therapy and temozolomide-based chemotherapy 1, 2. The prognosis of glioblastoma (GBM), anaplastic astrocytoma and other high-grade gliomas is extremely poor, leading to significant human mortalities each year 3, 4. Gliomas often contain heterogeneous mixture of cells exhibiting different cellular and nuclear polymorphism 5-10. In addition, dysregulation of multiple signaling cascades is essential for the uncontrolled growth and progression of glioma cells 3, 4, 11. Therefore, identifying novel glioma-driven signaling molecules and exploring the corresponding molecularly-targeted therapies are essential for better and efficient glioma therapy 3, 4, 11, 12. It has been the research focus of our group 13-15.

Dysfunctional mitochondria are commonly detected in human glioma, participating in tumorigenesis and cancer progression 16-18. In particular, aberrant energy metabolism, mitochondrial dysfunction, mitochondrial structural abnormalities and dysregulated mitochondrial apoptotic signalling cascade have been observed in malignant gliomas 16-18. Mitochondrial proteomics abnormalities could provide novel therapeutic targets for glioma 16-18. Our recent study has shown that overexpression of YME1L (YME1 Like 1 ATPase) 19-23, a mitochondrial inner membrane protease, exerts significant pro-tumorigenic activity in glioma 24.

TIMM44 (translocase of inner mitochondrial membrane 44) locates in the inner mitochondrial membrane 25, 26. In a ATP-dependent manner, TIMM44 anchors mitochondrial heat shock protein 70 to the translocase of the mitochondrial inner membrane 23 (TIMM23) complex 27-29. It promotes the import of mitochondrial preproteins to the mitochondrial matrix 27, 28. This process is dependent on inner membrane potential and ATP hydrolysis on ATPase domain of mtHsp70 27, 28. Wang *et al.,* have established an aP2-promoter driven TIMM44 transgenic mouse. The transgenic mice were protected from type 2 diabetes 25. Different mitochondrial fusion genes, including *mitofusin-1 (Mfn1), mitofusin-2 (Mfn2), and optic atrophy 1 (Opa1)*, were upregulated in TIMM44 transgenic mice 25. Overexpression of TIMM44 attenuated reactive oxygen species (ROS) production and increased ATP production, promoting human aortic smooth muscle cell proliferation 30.

Yu *et al.,* have shown that human antigen R directly associated with TIMM44 in ovarian cancer cells, vital for the *TIMM44* mRNA stability and ovarian cancer cell growth. Conversely, siRNA-induced silencing of TIMM44 inhibited ovarian cancer cell growth 31. Bonora *et al.,* have identified two novel variants in exon 9 and exon 13 of TIMM44, which could be associated with the development of oncocytic thyroid carcinomas 26. TIMM44 expression and potential functions in glioma have not been examined thus far. We here will show that TIMM44 overexpression exerts significant pro-tumorigenic activity in glioma.

## Results

### TIMM44 is overexpressed in GBM, correlating with poor survival

**Figure 1:**
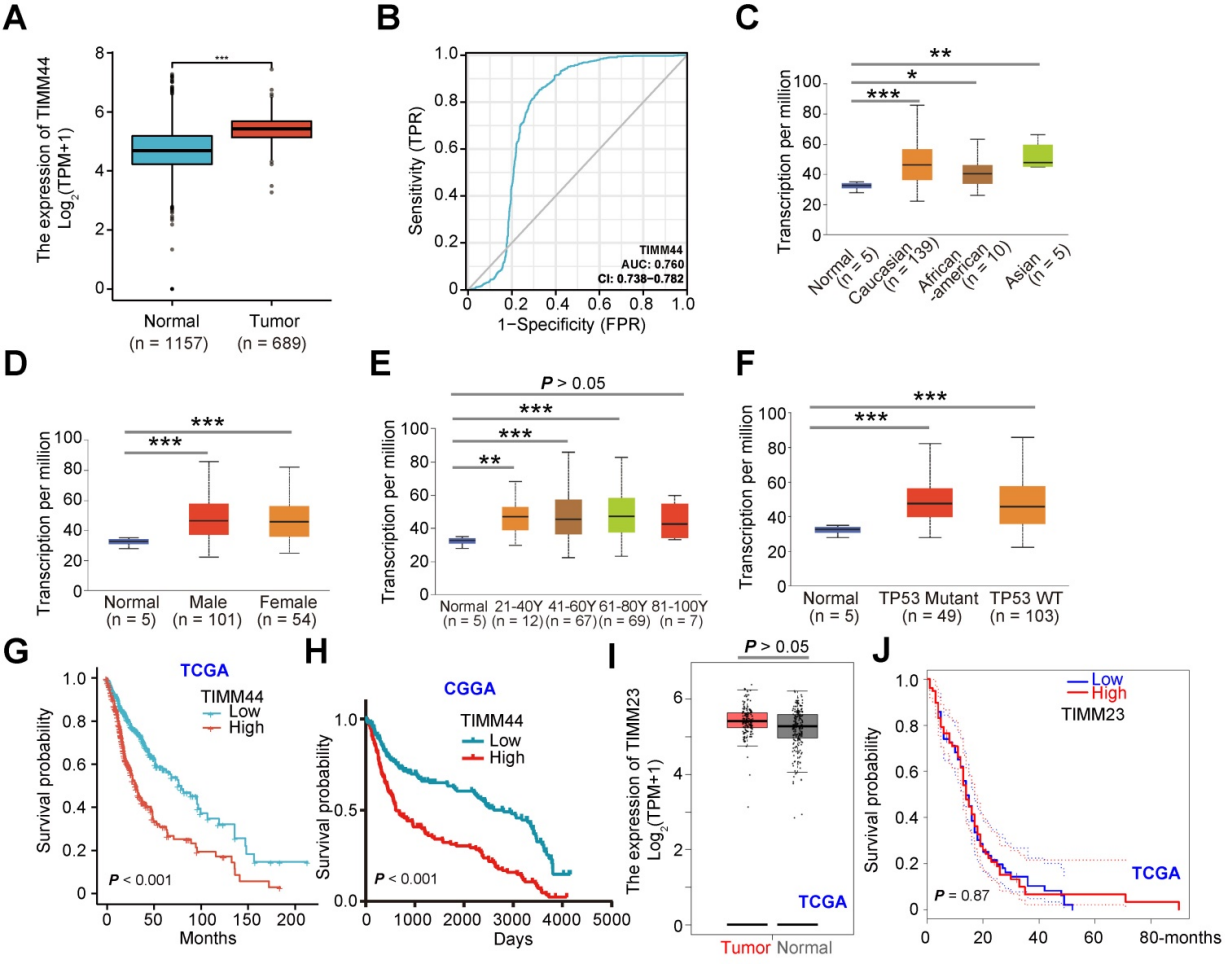
** TIMM44 is overexpressed in GBM, correlating with poor survival.** TCGA plus GTEx database shows *TIMM44* expression (RNA-Seq) in glioma tissues (“Tumor”, n = 689) and in the normal brain tissues (“Normal”, n = 1157) (**A**). The receiver operating characteristic (ROC) curve results demonstrated the relationship between *TIMM44* overexpression and the potential predictive effect on glioma patients' survival (**B**). The subgroup analyses of *TIMM44* mRNA expression and the listed clinical characteristics of glioma patients were shown (**C-F**). The Kaplan Meier Survival curves of *TIMM44*-low (in blue) and *TIMM44*-high (in red) GBM patients from The Cancer Genome Atlas (TCGA) (**G**) and Chinese Glioma Genome Atlas (CGGA) (**H**) databases were shown. TCGA database shows *TIMM23* expression (RNA-Seq) in glioma tissues (“Tumor”) and in the normal brain tissues (“Normal”) (**I**). The Kaplan Meier Survival curves of *TIMM23*-low (in blue) and *TIMM23*-high (in red) GBM patients from TCGA (**J**). “TPM” stands for transcripts per million. * *P* < 0.05. ** *P* < 0.01. *** *P* < 0.001.

We first analyzed *TIMM44* mRNA expression from The Cancer Genome Atlas (TCGA) database combining with the Genotype-Tissue Expression (GTEx) database. A total of 1846 human tissue samples were retrieved, of which 689 were GBM (“Tumor”), 1152 were normal brain tissues, and five were adjacent to cancer. The number of *TIMM44* transcripts in GBM tissues was significantly higher than that in the normal brain tissues (“Normal”) (Figure 1A). A receiver operating characteristic (ROC) curve is a plot of the sensitivity versus specificity for a diagnostic test. The different points on the curve indicate the different cut-points determining whether the test results are significant/positive 32. Area under curve (AUC) is recognized an effective method to summarize the overall diagnostic accuracy of the test. The ROC curve in Figure 1B showed the potential diagnostic value of TIMM44 for glioma. An AUC of 0.760 indicated an acceptable value of TIMM44 overexpression for potential diagnosis of glioma.

By consulting the UALCAN database (http://ualcan.path.uab.edu), the relationship between each subtype grouping and TIMM44 expression of 156 GBM tissues and five para-cancerous tissues was analyzed. *TIMM44* was highly expressed in different races (Figure 1C), both genders (Figure 1D) and different age groups (Figure 1E, except for the 81-100Y age). The high expression of *TIMM44* was however not correlated with p53 mutation status (Figure 1F). In both TCGA (Figure 1G) and Chinese Glioma Genome Atlas (CGGA) (Figure 1H), patients with high *TIMM44* expression in gliomas have worse prognosis and lower survival (Figure 1G and H). Whereas the patients with low *TIMM44* expression have better survival (Figure 1G and H). The bioinformatics results showed that TIMM44 is overexpressed in GBM, correlating with poor survival.

TIMM23 is another key component in the mitochondrial protein import machinery, binding to TIMM44 27-29. Interestingly, TCGA database results found that the number of *TIMM23* transcripts in GBM tissues was not upregulated when compared to that in the normal brain tissues (Figure 1I). Moreover, the overall survivals of high-*TIMM23* GBM patients and the low-*TIMM23* patients were equivalent (*P* = 0.87, Figure 1J).

### Mitochondrial TIMM44 overexpression in local glioma tissues and cells

**Figure 2:**
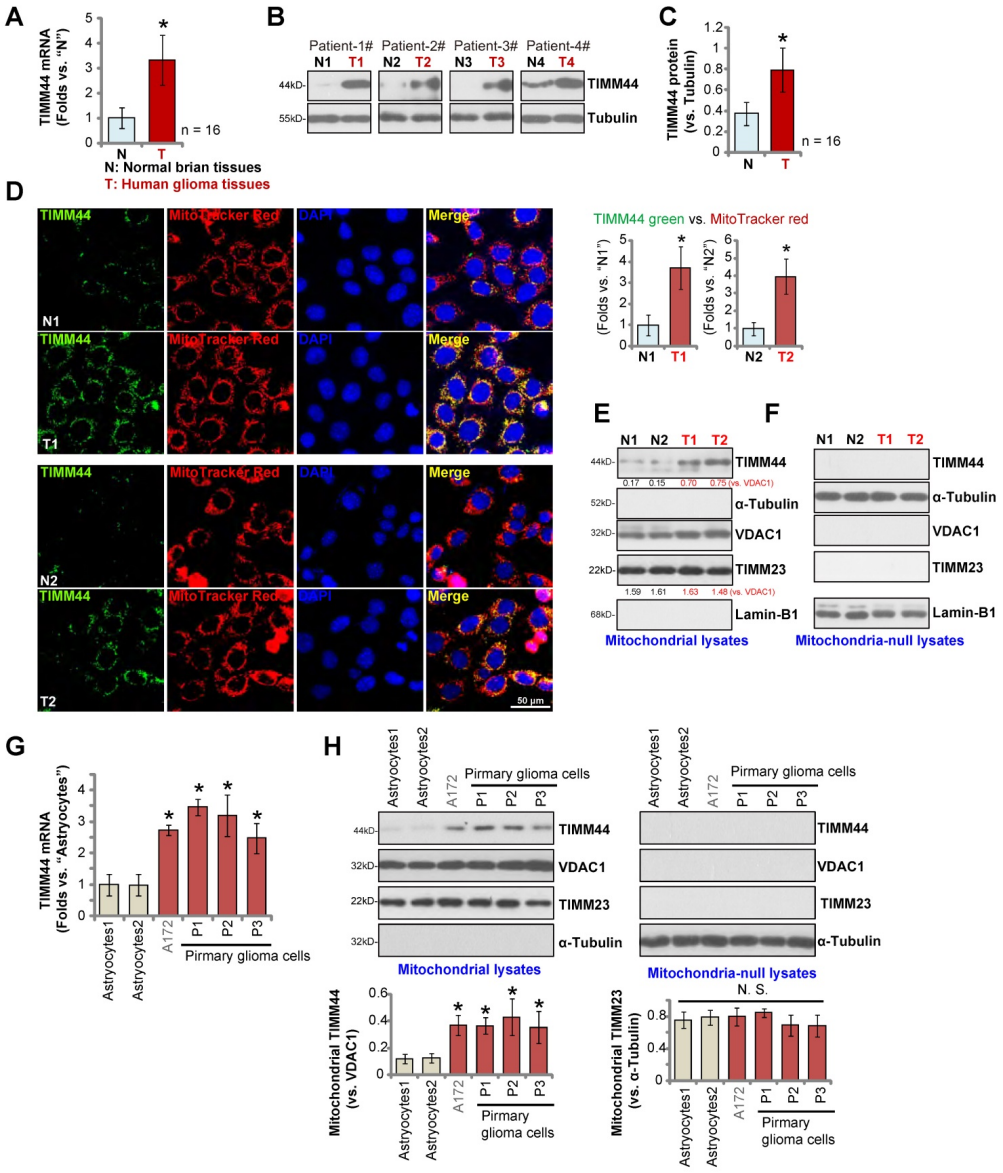
** Mitochondrial TIMM44 overexpression in local glioma tissues and cells.** The human glioma tissues (“T”) and the paired adjacent normal brain tissues (“N”), derived from a total of sixteen (16) HGG patients, were homogenized, *TIMM44 mRNA* and protein expression in tissue lysates was examined by qRT-PCR (**A**) and Western blotting (**B** and **C**) assays, respectively. The human tissue immuno-fluorescence images showed TIMM44 protein (green fluorescence), MitoTracker (red fluorescence), and DAPI (in the red fluorescence) in glioma tissue slides (“T1/T2”) and the adjacent normal brain tissue slides (“N1/N2”) of two representative glioma patients (Patient-1# and Patient-2#) (**D**). TIMM44 green fluorescence intensity was recorded from 10 random microscopy views of each slide, and its intensity was normalized to MitoTracker red fluorescence intensity (**D**, the right panel). Mitochondrial lysates and mitochondria-null lysates of the two representative glioma patients (Patient-1# and Patient-2#) were observed and listed proteins were tested (**E** and **F**). *TIMM44 mRNA* (**G**) and listed proteins (in mitochondrial and mitochondrial-null lysates, **H**) expression in the primary human astrocytes (“Astrocytes1/2”, derived from two patients), the immortalized (A172) and primary (P1, P2 and P3) glioma cells was shown. The data were presented as mean ± standard deviation (SD).* ***P*** < 0.05 *vs.*“N”/“Astrocytes”.

We also examined TIMM44 expression in local glioma tissues. A total of sixteen (n = 16) pairs of high-grade (grade III-IV) glioma tissues (“T”) and matched adjacent normal brain tissues (“N”), as reported in our previous studies 13, 15, 24, were analyzed. qRT-PCR results, Figure 2A, demonstrated that *TIMM44* mRNA expression in the glioma tissues was significantly higher than that in the normal brain tissues. Western blotting data of four representative patients, Patient-1 to Patient-4, confirmed TIMM44 protein upregulation in glioma tissues (Figure 2B). Combining all 16 pairs of blotting data showed that TIMM44 protein upregulation in glioma tissues was significant (*P* < 0.05 vs. “N” tissues) (Figure 2C).

The tissue immuno-fluorescence images in Figure 2D demonstrated that TIMM44 protein (labeled in the green fluorescence) was co-localized with the MitoTracker (red fluorescence) in both glioma slides and adjacent normal brain slides of two representative patients (Patient-1# and Patient-2#). TIMM44 green fluorescence intensity in glioma slides was higher than that in the matched normal brain slides (Figure 2D). TIMM44 green fluorescence intensity was recorded from 10 random microscopy views (containing 14-15 nuclei per view) of each slide, and intensity normalized to MitoTracker red fluorescence intensity. As shown, the normalized TIMM44 green fluorescence intensity in the glioma slides was dramatically higher than that in the matched normal brain slides (Figure 2D, the right panel).

Furthermore, the mitochondrial fractions of human tissues of the two representative patients (Patient-1# and Patient-2#) were isolated and tissue lysates were analyzed. TIMM44 protein was only enriched in the mitochondrial lysates (Figure 2E), as indicated by the mitochondrial marker protein VDAC1 (voltage-dependent anion-selective channel 1) (Figure 2E). Both the nuclear marker protein Lamin-B1 and the cytosol marker protein α-Tubulin were not present in the mitochondrial fraction lysates (Figure 2E). Notably, the mitochondrial TIMM44 expression was higher in the glioma tissues (Figure 2E). TIMM23 protein levels were indifferent between glioma tissues and normal brain tissues (Figure 2E). TIMM44, TIMM23 and VDAC1 proteins were not detected in the mitochondria-null lysates of the human tissues (Figure 2F), whereas Lamin-B1 and α-Tubulin were present (Figure 2F). Thus, mitochondria-localized TIMM44 is upregulated in human glioma tissues.

We also examined TIMM44 expression in human glioma cells. Patient-derived primary glioma cells “P1”, “P2” and “P3” (derived from three patients 13) and the immortalized cell line (A172) were examined. Results showed that *TIMM44* mRNA expression in the primary and established glioma cells was higher than that in the primary human astrocytes (“Astrocytes1/2”, derived from two patients, Figure 2G). Moreover, TIMM44 protein upregulation was observed (Figure 2H). TIMM23 and VDAC1 protein expression was equivalent between astrocytes and glioma cells (Figure 2H). The relative expression of mitochondrial TIMM44 protein, after normalization to the mitochondrial proteinVDAC1, was again increased in different glioma cells (Figure 2H). These results confirmed mitochondrial TIMM44 overexpression in local glioma tissues and cells.

### TIMM44 depletion induces significant anti-glioma cell activity

**Figure 3:**
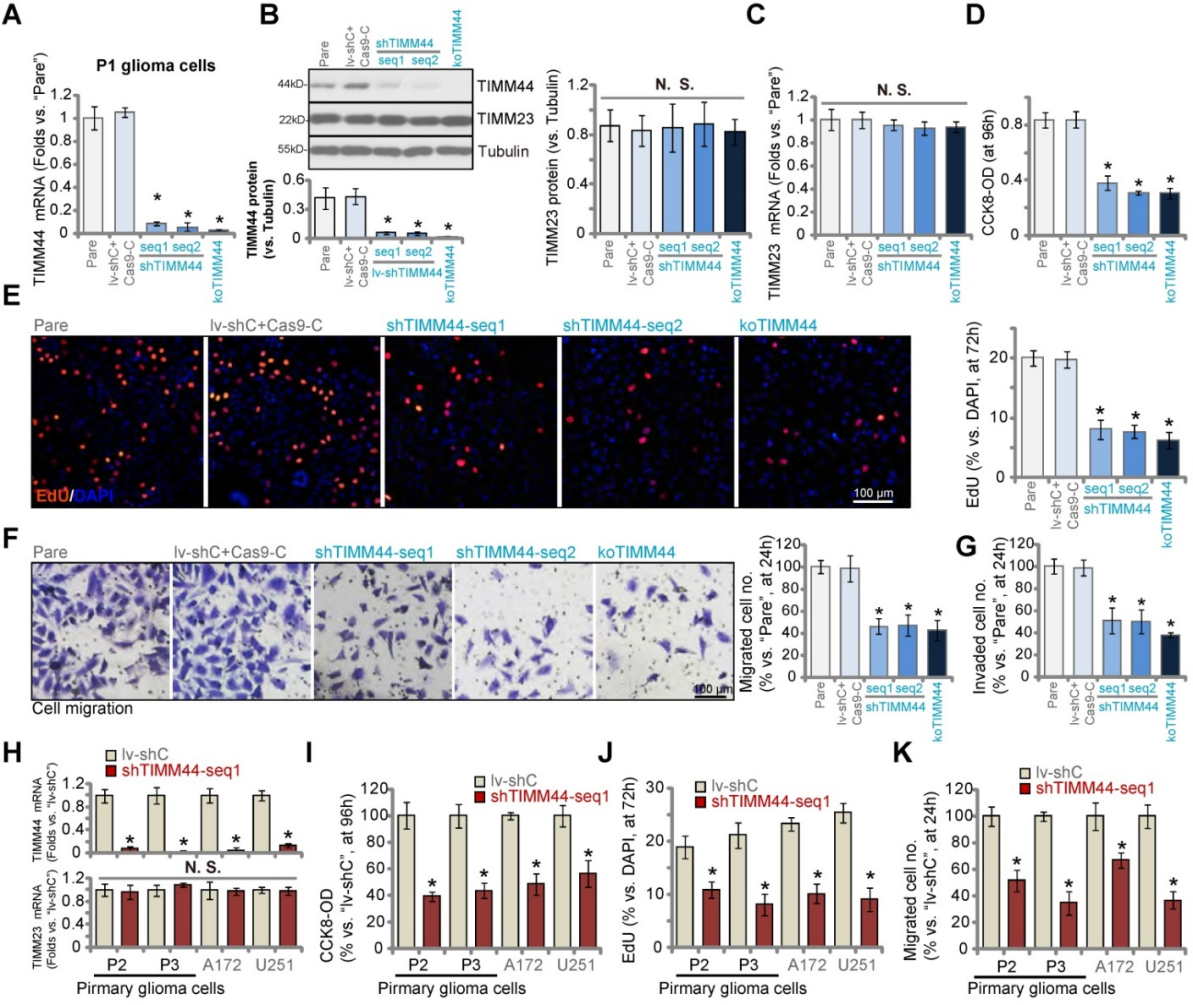
** TIMM44 depletion induces significant anti-glioma cell activity.** The primary human glioma cells, P1, stably expressing the listed TIMM44 shRNA (shTIMM44-seq1/shTIMM44-seq2), the Cas9 construct plus the CRISPR/Cas9-TIMM44-KO construct (“koTIMM44”), or the lentiviral scramble control shRNA plus the Cas9 empty vector (“lv-shC+Cas9-C”), were formed and were tested for the listed genes and proteins (**A**-**C**). Alternatively, cells were further cultivated for designated hours, and cellular functions, including cell viability (**D**), proliferation (**E**), migration (**F**) and invasion (**G**) were examined. Expression of listed mRNAs in the primary glioma cells (“P2” and “P3”) or established lines (A172 and U251) with the TIMM44 shRNA (shTIMM44-seq1) or the lentiviral scramble control shRNA (“lv-shC”) was shown (**H**). The glioma cells were further cultivated for designated hours, and cell viability (**I**), proliferation (**J**) and migration (**K**) were tested, with the results quantified. “Pare” stands for the parental control glioma cells. The data were presented as mean ± standard deviation (SD). * ***P*** < 0.05 *vs.* “Pare”/“lv-shC”. “N. S.” stands for non-statistical difference (***P*** > 0.05). The *in vitro* experiments were repeated five times with similar results obtained. Scale bar = 100 µm.

Using the previously described genetic methods 13, 15, 24, we depleted TIMM44 in human glioma cells. Specifically, the lentivirus encoding the TIMM44 shRNA (shTIMM44-seq1/shTIMM44-seq2, with non-overlapping sequences) was added to P1 primary human glioma cells 13, 15, 24. Stable cells were formed following selection through puromycin-containing medium. Alternatively, a CRISPR/Cas9-TIMM44-KO vector was established and transduced to the Cas9-expressing P1 glioma cells 24. The transfected cells were thereafter distributed into 96-well plates. Through *TIMM44* KO screening, stable TIMM44-KO cells were formed. These cells were named as “koTIMM44” cells. As shown *TIMM44* mRNA levels were dramatically decreased in shTIMM44 and ko-TIMM44 P1 glioma cells (Figure 3A). The applied shRNAs and KO construct resulted in TIMM44 protein depletion as well in P1 glioma cells (Figure 3B). *TIMM23* mRNA and protein expression was unchanged (Figure 3B and C).

TIMM44 shRNA or KO significantly decreased CCK-8 viability in P1 primary glioma cells (Figure 3D). Moreover, the percentage of EdU-positive nuclei, an indicator of cell proliferation, was dramatically decreased in shTIMM44-expressing P1 glioma cells and koTIMM44 cells (Figure 3E). Moreover, TIMM44 shRNA or KO largely suppressed P1 glioma cell *in vitro* migration (Figure 3F) and invasion (Figure 3G), tested by “Transwell” and “Matrigel Transwell” assays, respectively. The control P1 glioma cells were with the lentiviral scramble shRNA plus the CRSIPR/Cas9 empty vector (“lv-shC+Cas9-C”), which failed to alter TIMM44-TIMM23 expression (Figure 3A-C) and glioma cell functions (Figure 3D-G).

MB-10 (“MitoBloCK-10”) binds to a specific pocket in the C-terminal domain of TIMM44, thereby inhibiting mitochondrial protein import 33. Here we found that treatment with MB-10 in P1 primary human glioma cells robustly inhibited CCK-8 cell viability (Figure S1A) and proliferation (EdU-nuclei ratio reduction, Figure S1B). P1 cell migration (Figure S1C) and invasion (Figure S1D) were largely slowed following MB-10 treatment as well.

Other primary human glioma cells (“P2” and “P3”, derived from other patients 13, 15) and established cell lines (A172 and U251) were infected with shTIMM44-seq1-expressing lentivirus, and puromycin was added to select stable cells. As compared to control glioma cells with the lentiviral scramble shRNA (lv-shC), TIMM44 expression was robustly decreased in shTIMM44-seq1-expressing glioma cells (Figure 3H). The viability of the primary and established glioma cells was decreased following TIMM44 silencing (Figure 3I), which also exerted anti-proliferative activity (Figure 3J) and hindered *in vitro* migration (Figure 3K) of the glioma cells.

The primary human astrocytes, Astrocytes1 and Astrocyte2, were stably infected with the shTIMM44-seq1-expressing lentivirus, leading to downregulation of *TIMM44* mRNA (Figure S2A). *TIMM23* mRNA expression was unchanged (Figure S2B). However, in the astrocytes, TIMM44 silencing failed to significantly inhibit cell viability (Figure S2C) and proliferation (Figure S2D).

### The mitochondrial functions are disrupted with TIMM44 depletion in glioma cells

**Figure 4:**
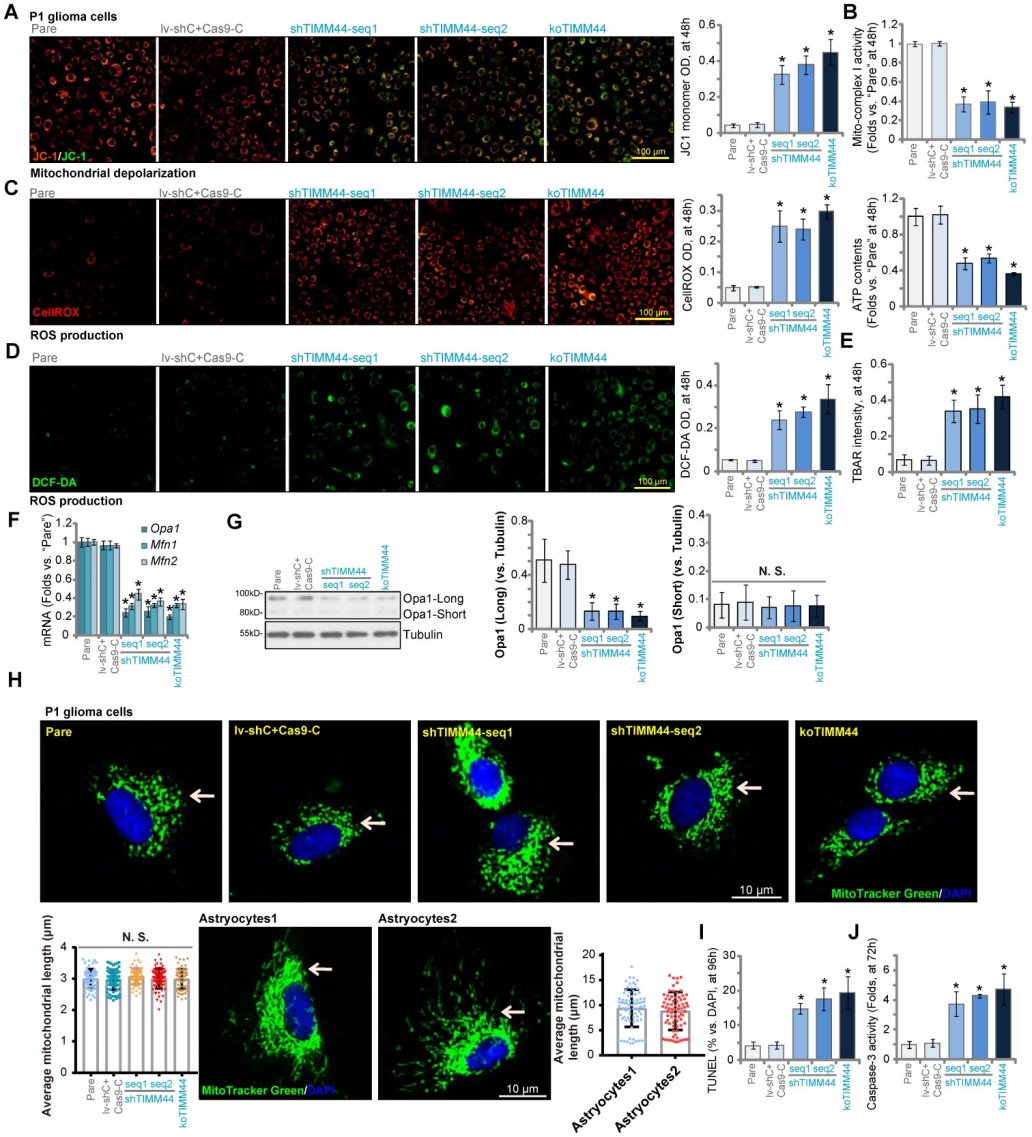
** The mitochondrial functions are disrupted with TIMM44 depletion in glioma cells.** P1 glioma cells with applied genetic modifications were cultivated for designated hours, mitochondrial membrane potential (**A**), the relative mitochondrial complex I activity and cellular ATP contents (**B**), ROS intensity (DCF-DA/CellROX OD, **C** and **D**) and lipid peroxidation (TBAR activity, **E**) were examined by the assays mentioned. mRNA and listed proteins were tested (**F** and **G**). The high-resolution fluorescence images showing mitochondrial fusion/fission morphology in the P1 glioma cells and the primary astrocytes were presented as well (**H**), and average mitochondrial length was quantified (**H**). Cell apoptosis (by testing TUNEL-nuclei ratio, **I**) and relative Caspase-3 activity (**J**) were examined. “Pare” stands for the parental control glioma cells. The data were presented as mean ± standard deviation (SD). * ***P*** < 0.05 *vs.* “Pare”. “N. S.” stands for non-statistical difference (***P*** > 0.05). The *in vitro* experiments were repeated five times with similar results obtained. Scale bar = 10 µm /100 µm.

Studies have shown that increasing TIMM44 expression can restore mitochondrial functions 25. We therefore analyzed whether TIMM44 depletion could result in mitochondrial dysfunctions in human glioma cells. As shown, in P1 glioma cells TIMM44 shRNA or KO (see Figure 3) induced conversion of JC-1 aggregates (in red fluorescence) to the JC-1 green monomers (Figure 4A), causing mitochondrial depolarization. Moreover, the mitochondrial complex I activity and the cellular ATP contents were significantly decreased following TIMM44 silencing or depletion in P1 glioma cells (Figure 4B). Further studies showed that cellular ROS contents were significantly increased in P1 cells with TIMM4 shRNA or KO, and the DCF-DA green intensity (Figure 4C) and the CellROX red intensity (Figure 4D) were both increased. Supporting increased lipid peroxidation, we found that the TBAR activity was augmented in TIMM44-silenced or TIMM44-KO P1 glioma cells (Figure 4E). TIMM44 shRNA (by shTIMM44-seq1) or KO robustly inhibited the import of Su9-DHFR fusion protein into the mitochondria of P1 glioma cells (Figure S4A), and about 60-70% reduction of mitochondrial protein import was achieved with TIMM44 silencing/KO (Figure S4A).

Treatment with the mitochondrial protein import blocker MB-10 induced mitochondrial depolarization (JC-1 green monomers accumulation, Figure S1E) and ROS production (CellROX intensity increasing, Figure S1F). Significant apoptosis activation, evidenced by TUNEL-positive nuclei ratio increasing, was observed as well in MB-10-treated P1 glioma cells (Figure S1G).

mRNA expression of TIMM44-dependent mitochondrial genes, including *Opa1*, *Mfn1* and *Mfn2* 25, were dramatically decreased with TIMM44 silencing or KO in P1 glioma cells (Figure 4F). TIMM44 shRNA or KO also decreased the expression of OPA1 protein (the long and un-cleaved form, Figure 4G), but did not alter OPA1 protein cleavage, as the short and cleaved form of OPA1 was unchanged (Figure 4G). Cancer cells have increased mitochondrial fission to achieve proliferative and survival advantages 34-36. The high-resolution mitochondrial fluorescence images clearly showed that the majority of mitochondria in the parental control P1 glioma cells were small-fragment fission mitochondria (Figure 4H). The average mitochondrial length was quantified from 25 mitochondria per cell of three different cells for each condition. We found that the average mitochondrial length was similar between control glioma cells and TIMM44-silenced/KO glioma cells (Figure 4H). On the contrast, a significant number of fused mitochondria (long fragment) were detected in the non-cancerous primary human astrocytes (“Astrocytes1/2”) (Figure 4H), showing longer average mitochondrial length (Figure 4H).

With mitochondrial dysfunction, apoptosis was induced in TIMM44-depeleted glioma cells. In TIMM44-silenced or TIMM44-KO P1 glioma cells, TUNEL-positive nuclei percentage was significantly increased (Figure 4I). Moreover, the relative Caspase-3 activity was augmented (Figure 4J). Supplement ATP or adding the antioxidant NAC robustly ameliorated TIMM44 silencing (by shTIMM44-seq1)-induced viability (CCK-8 OD) reduction and cell death (Trypan blue staining) in P1 glioma cells (Figure S3A and B), suggesting that mitochondrial dysfunction should be the primary cause of TIMM44-depeletion-induced cytotoxicity in glioma cells.

Similar to our results in P1 glioma cells, we found that TIMM44 shRNA decreased mitochondrial complex I activity (Figure S3C) and ATP contents (Figure S3D), while increasing the ROS levels (CellROX intensity increase, Figure S3E) in P2/ P3 primary glioma cells and established lines (A172 and U251). TIMM44 shRNA also increased the number of TUNEL-positive nuclei in the glioma cells (Figure S3F).

In the primary human astrocytes, Astrocytes1 and Astrocyte2, TIMM44 shRNA failed to significantly alter ATP contents (Figure S4B) and ROS levels (CellROX intensity, Figure S4C). Moreover, it failed to increase the Caspase-3 activity (Figure S4D) and provoke apoptosis (Figure S4E).

### The pro-cancerous activity by ectopic TIMM44 expression in glioma cells

**Figure 5:**
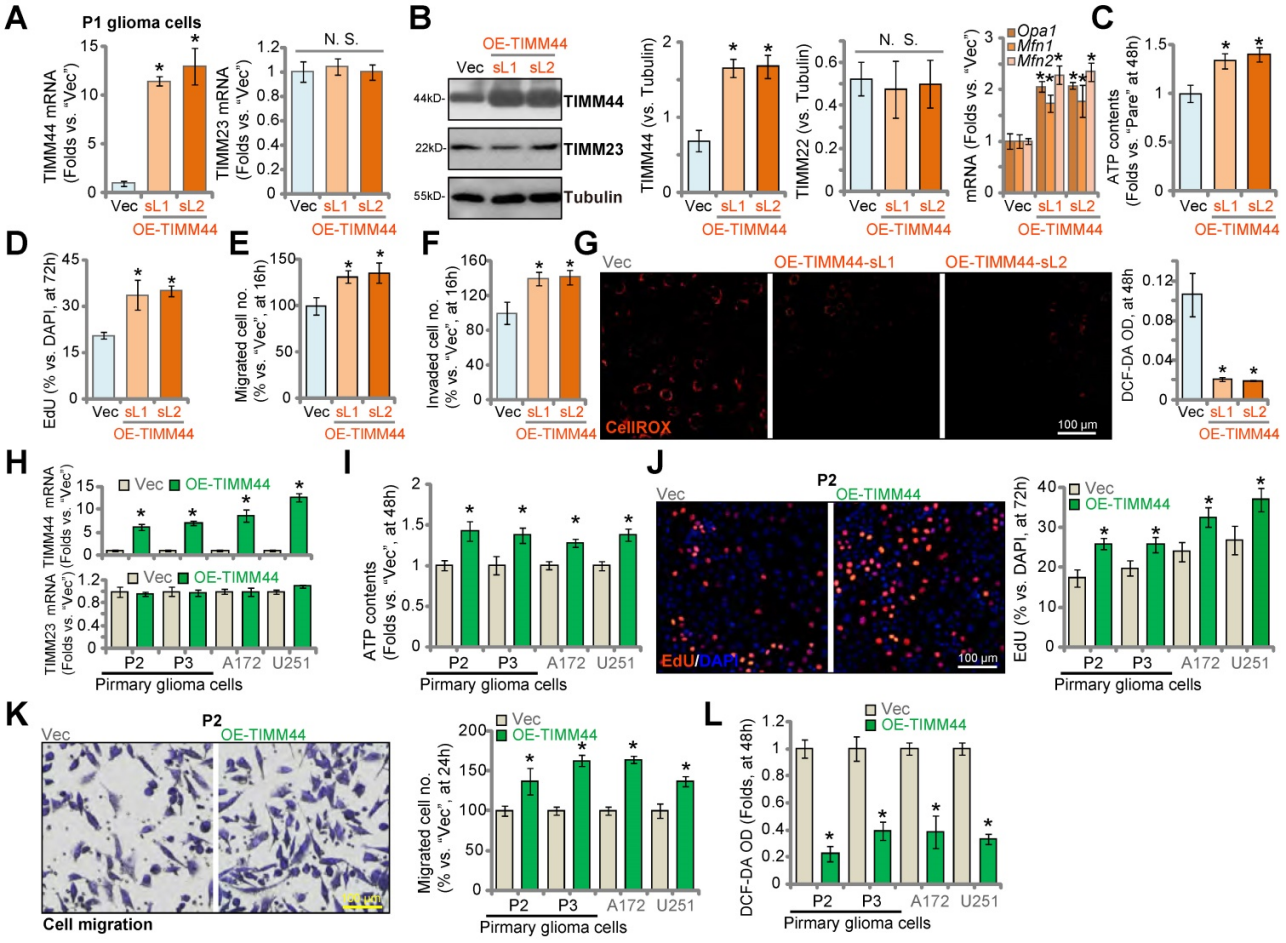
** The pro-cancerous activity by ectopic TIMM44 expression in glioma cells.** The primary human glioma cells, P1, stably expressing the lentiviral TIMM44-expressing vector (OE-TIMM44-sL1/OE-TIMM44-sL2, representing two stable selections) or the empty vector (“Vec”), were formed and tested for the listed genes and proteins (**A** and **B**). The glioma cells were further cultivated for designated hours, ATP contents were measured (**C**); EdU-positive nuclei ratio (**D**), migrated cell number (**E**), invaded cell number (**F**) and CellROX intensify (**G**) were measured, with results quantified. Expression of listed mRNAs in the primary glioma cells (“P2” and “P3”) or established lines (A172 and U251) with the lentiviral TIMM44-expressing vector (“OE-TIMM44”) or the empty vector (“Vec”) was shown (**H**). The glioma cells were further cultivated for designated hours, and ATP contents (**I**), proliferation (EdU-positive nuclei ratio, **J**), migration (**K**) and CellROX intensity (**L**) were tested. The data were presented as mean ± standard deviation (SD). * ***P*** < 0.05 *vs.* “Vec”. “N. S.” stands for non-statistical difference (***P*** > 0.05). The *in vitro* experiments were repeated five times with similar results obtained. Scale bar = 100 µm.

Thus far, we found that genetic depletion of TIMM44 inhibited cell proliferation and migration, disrupted mitochondrial functions and induced apoptosis in primary and established glioma cells. We therefore tested whether ectopic overexpression of TIMM44 could exert pro-glioma cell activity. To this purpose, the lentiviral TIMM44-expressing vector were transduced to P1 primary glioma cells, and puromycin was utilized to select stable cells: OE-TIMM44-sL1 and OE-TIMM44-sL2 (representing two different stable selections). In OE-TIMM44 P1 glioma cells *TIMM44* mRNA and protein levels were indeed dramatically increased (Figure 5A and B) and TIMM23 expression was unchanged (Figure 5A and B). *Opa1*, *Mfn1* and *Mfn2*, TIMM44-dependent mitochondrial genes, were increased in TIMM44-overexpressed P1 glioma cells (Figure 5B). In OE-TIMM44 cells, the cellular ATP contents were significantly increased (Figure 5C). Ectopic overexpression of TIMM44 promoted P1 glioma cell proliferation, which was evidenced by the increased EdU-positive nuclei percentage (Figure 5D). Moreover, enhanced numbers of migrated cells (Figure 5E) and invaded cells (Figure 5F) were observed after TIMM44 overexpression. On the contrast, DCF-DA intensity was decreased (Figure 5G), suggesting decreased ROS contents following TIMM44 overexpression in primary glioma cells.

To P2 and P3 primary glioma cells and established cell lines (A172 and U251), the TIMM44-expressing lentivirus was added, and stable cells were formed (“OE-TIMM44”). As compared to the vector control cells (“Vec”), *TIMM44* mRNA, but not *TIMM23* mRNA, was increased in OE-TIMM44 glioma cells (Figure 5H). The cellular ATP contents were augmented in the TIMM44-overexpressed glioma cells (Figure 5I). TIMM44 overexpression potentiated cell proliferation (increasing EdU-positive nuclei percentage, Figure 5J) and migration (Figure 5K) in the glioma cells. While the cellular ROS contents, or the DCF-DA intensity, were decreased (Figure 5L). These results further supported the essential role of TIMM44 in the progression of glioma cells.

The lentiviral TIMM44-expressing vector was also transduced to the primary human astrocytes (Astrocytes1 and Astrocyte2), resulting in significant *TIMM44* upregulation (“OE-TIMM44”, Figure S5A). *TIMM23* mRNA levels were unchanged (Figure S5B). OE-TIMM44 had no significant effect on cell viability (CCK-8 OD, Figure S5C) and proliferation (by measuring EdU-positive nuclei ratio, Figure S5D) in human astrocytes. These results again supported the cancer cell-specific effect by TIMM44.

### YME1L promotes GATA3-dependent *TIMM44* transcription in glioma cells

Our previous study has shown that YME1L (YME1 Like 1 ATPase), a mitochondrial inner membrane protease, is upregulated in human glioma, and it is essential for glioma cell growth *in vitro* and *in vivo* 24. YME1L shRNA or KO inhibited glioma cell viability, proliferation and migration, and induced apoptosis activation 24. TCGA database retrieving differentially expressed genes (DEGs) with *YME1L* in human glioma tissues and the following Pearson Correlation Coefficient analyses showed that *TIMM44* is one topYME1L's DEG with the second highest correlation score 24.

**Figure 6:**
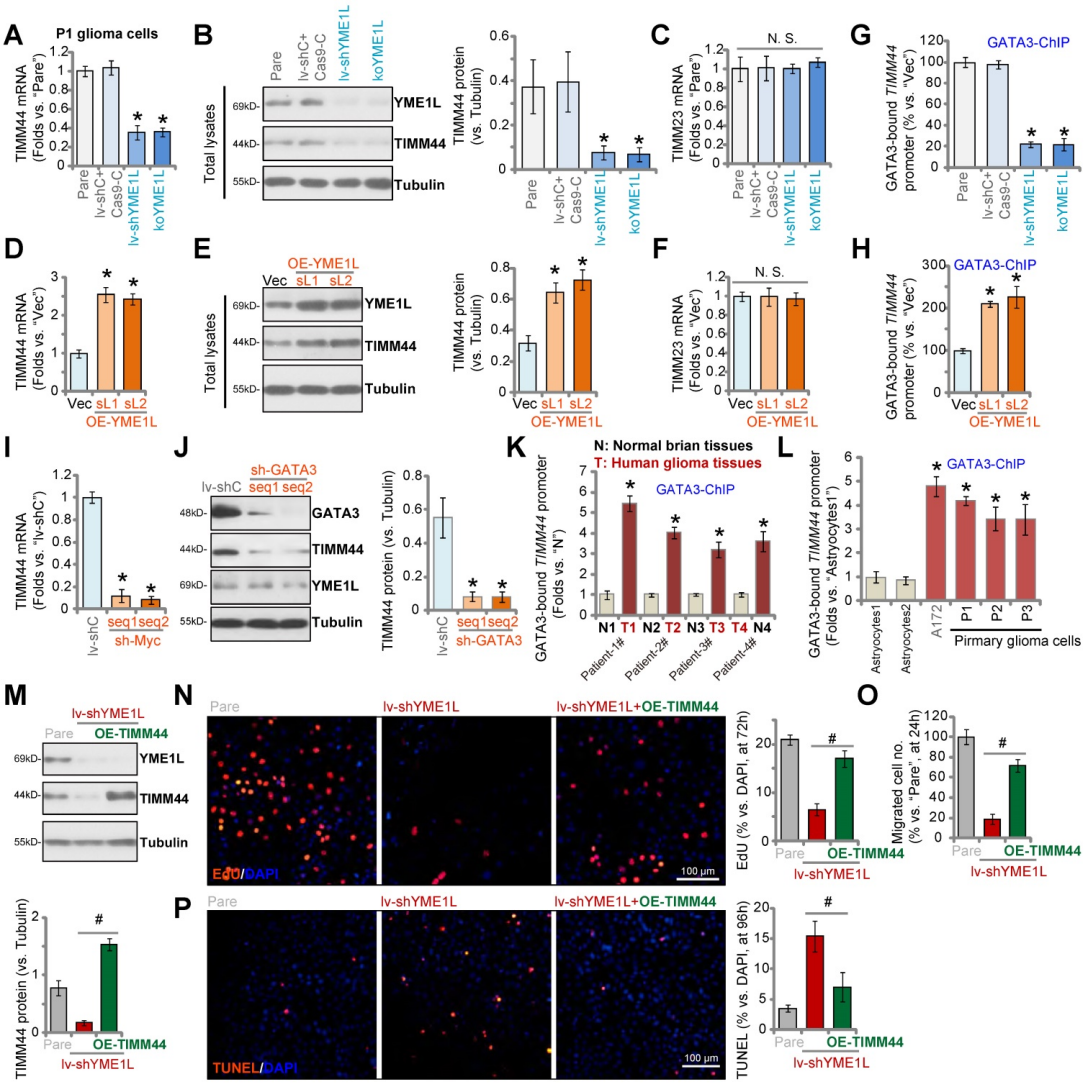
** YME1L promotes GATA3-dependent TIMM44 transcription in glioma cells.** P1 primary human glioma cells, stably expressing the YME1L shRNA (lv-shYME1L), the lenti-CRSIPR/Cas9-YME1L-KO-puro construct (“koYME1L”) or the lentiviral scramble shRNA plus the CRSIPR/Cas9 empty vector (“lv-shC+Cas9-C”), were established, expression of listed genes and proteins (in total cell lysates and nuclear lysates) was shown (**A**-**C**). P1 glioma cells with the YME1L-expressing construct (“OE-YME1L-sL1 or OE-YME1L-sL2”, two stable selections) or the empty vector (“Vec”) were established, and the expression of listed genes and proteins was shown (**D-F**). Chromosome IP (ChIP) showed the relative levels of the proposed *TIMM44* promoter binding to GATA3 in the P1 glioma cells with genetic modifications (**G** and **H**), or in the listed human tissue lysates (**K**) and astrocytes/glioma cells (**L**). P1 glioma cells expressing the scramble control shRNA (lv-shC), the lentiviral GATA3 shRNA (“sh-GATA3-seq1/2”, two different sequences) were established, expression of listed genes and protein was shown (**I** and **J**). The lv-shYME1L P1 glioma cells were further transduced with or without the lentiviral TIMM44-expressing vector (“OE-TIMM44”), expression of the listed proteins was shown (**M**). Cells were further cultivated for the indicated time periods, cell proliferation, migration and apoptosis were tested by EdU staining (**N**), “Transwell” (**O**), and TUNEL staining (**P**) assays, respectively. The data were presented as mean ± standard deviation (SD). * ***P*** < 0.05 *vs.* “Pare”/“Vec”/“lv-shC”/“N” tissues/“Astryocytes1”. ^#^ ***P*** < 0.05. “N. S.” stands for non-statistical difference (***P*** > 0.05). The *in vitro* experiments were repeated five times with similar results obtained. Scale bar = 100 µm.

Next, YMEL1 shRNA-expressing lentivirus was transfected to P1 primary human glioma cells and stable cells (“lv-shYME1L”) were formed by selection. Alternatively, a lenti-CRSIPR/Cas9-YME1L-knockout (KO)-puro construct was transduced to Cas9-expressing P1 glioma cells, and single stable cells (“koYME1L”) were established following selection and YMEL1 KO screening. As shown YME1L shRNA and KO resulted in significant *TIMM44 mRNA* (Figure 6A) and protein (Figure 6B) downregulation in P1 glioma cells. YME1L protein was depleted as well (Figure 6B). Expression of *TIMM23* mRNA was unchanged (Figure 6C). YME1L silencing or KO resulted in reduction of TIMM44-dependent mitochondrial genes, including *Opa1*, *Mfn1* and *Mfn2*, in P1 glioma cells (Figure S6A).

In contrast, the lentiviral YME1L-expressing construct was transduced to P1 glioma cells. Two stable selections, OE-YME1L-sL1 and OE-YME1L-sL2 24, were formed after selection using puromycin. We found that *TIMM44* mRNA (Figure 6D) and protein (Figure 6E) as well as the YME1L protein (Figure 6E) levels were significantly increased in OE-YME1L glioma cells, whereas* TIMM23* mRNA was unchanged (Figure 6F). *Opa1*, *Mfn1* and *Mfn2* mRNA levels were increased as well in YEM1L-overexpressed cells (Figure S6B). These results indicated that YME1L is important for TIMM44 expression in primary glioma cells.

Since YME1L is an i-AAA protease but not a transcriptional regulator, it is unlikely that YME1L can directly regulate TIMM44 at mRNA level. We therefore explored the transcriptional mechanism of TIMM44 expression in glioma cells. The tfbind database (https://tfbind.hgc.jp/) predicted transcription factors with the highest binding affinity to *TIMM44* promoter. siRNAs specifically targeting these transcription factors were individually transfected to P1 primary glioma cells. Among the tested siRNAs, only GATA3 (GATA binding protein 3) siRNA resulted in robust *TIMM44* mRNA downregulation. Importantly, GATA3 chromosome immunoprecipitation (ChIP) results demonstrated that GATA3-*TIMM44* promoter binding in P1 glioma cells was robustly decreased after YME1L silencing or depletion (Figure 6G). Whereas overexpression of YME1L increased GATA3-*TIMM44* promoter binding (Figure 6H).

shRNA-induced silencing of GATA3 significantly decreased *TIMM44* mRNA and protein expression in P1 glioma cells (Figure 6I and J), and YME1L expression was unchanged (Figure 6J). Moreover, in human glioma tissues of four representative patients, GATA3 binding to the *TIMM44* promoter was significantly higher than that in the matched surrounding normal brain tissues (Figure 6K). In addition, GATA3-*TIMM44* promoter binding was augmented in different glioma cells (Figure 6L). Therefore, YME1L promoted GATA3-dependent *TIMM44* transcription in glioma cells. ChIP results demonstrated that the YME1L shRNA-induced decrease of GATA3-*TIMM44* promoter binding was attenuated by exogenously adding ATP in P1 glioma cells (Figure S6C). ATP also partially restored *TIMM44* mRNA (Figure S6D) and protein (Figure S6E) expression in YME1L-silenced glioma cells.

Moreover, overexpression of YME1L, by the lentiviral YME1L-expressing construct (“+OE-YME1L”), failed to restore TIMM44 *mRNA* expression in GATA3-silenced P1 glioma cells (Figure S6F). Therefore, *TIMM44* downregulation in YME1L-deficient glioma cells should be caused by shutdown of GATA3-dependent* TIMM44* transcriptional machinery following mitochondrial stress.

Next, the lentiviral TIMM44-expressing construct (“OE-TIMM44”) was transduced to the lv-shYME1L P1 glioma cells and restored TIMM44 expression (Figure 6M). YME1L silencing inhibited P1 glioma cell proliferation (Figure 6N) and migration (Figure 6O), and provoked apoptosis (Figure 6P). Remarkably, OE-TIMM44 inhibited shYME1L-induced anti-glioma cell activity (Figure 6N-P), partially restoring cell proliferation (Figure 6N) and migration (Figure 6O), and alleviating cell apoptosis (Figure 6P).

### TIMM44 depletion inhibits subcutaneous and intracranial glioma xenograft growth *in vivo*

At last we tested the potential effect of TIMM44 on glioma cell growth *in vivo*. P1 glioma cells were inoculated and subcutaneously (s.c.) injected to the flanks of the nude mice. The subcutaneous P1 glioma xenografts were formed three weeks after cell injection with tumor volumes close to 100 mm^3^ (“Day-0”). The xenograft-bearing mice were then randomly separated into two different groups. The treatment group received intratumoral injection of TIMM44-shRNA AAV (“aav-shTIMM44”, 10 μL virus per tumor, 1×10^9^ PFU), and the control group received intratumoral injection of the same amount of scramble control shRNA AAV (“aav-shC”). In each group there were ten mice (n = 10), and the AAV was injected every 24h for ten rounds. The xenograft tumor volumes were recorded every five days, from “Day-0” to “Day-35” (seven rounds).

**Figure 7:**
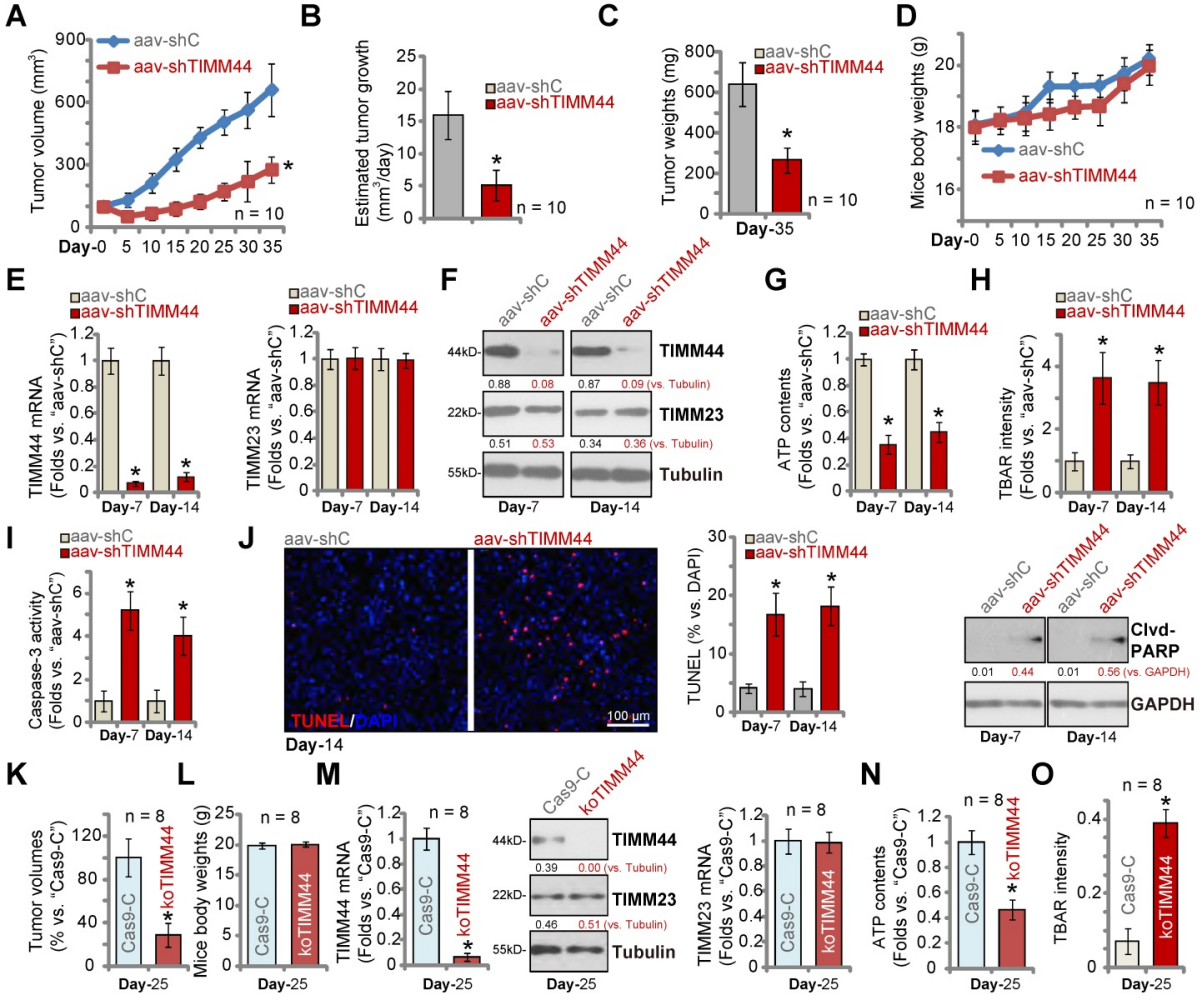
** TIMM44 depletion inhibits subcutaneous and intracranial glioma xenograft growth *in vivo*.** The subcutaneous P1 glioma xenograft-bearing mice were subject to intratumoral injection of TIMM44-shRNA AAV (“aav-shTIMM44”) or the scramble control shRNA AAV (“aav-shC”). Virus was injected daily for 10 days. The xenograft tumor volumes (**A**) and mice body weights (**D**) were recorded every five days. The estimated daily tumor growth (in mm^3^ per day) (**B**) was calculated. At Day-35 all tumors were isolated and weighted (**C**). The listed tumors were homogenized, and expression of listed genes and proteins was tested (**E** and **F**). ATP contents (**G**) and TBAR activity (**H**) were analyzed in the tissue lysates. Apoptosis intensity and expression of listed proteins were analyzed by the listed assays (**I** and **J**). P1 primary human glioma cells (5 × 10^5^ cells of each mouse), with the lenti-CRSIPR/Cas9-TIMM44-KO-puro construct (koTIMM44) or control construct (“Cas9-C”), were intracranially injected to brains of nude mice; After 25 days (“Day-25”), animals were decapitated and tumors were isolated, the tumor volumes (**K**) and mice body weights were recorded (**L**). Expression of listed genes and proteins was tested by qRT-PCR and Western blotting assays (**M**); ATP contents (**N**) and the TBAR activity (**O**) were measured as well. The data were presented as mean ± standard deviation (SD). * ***P*** < 0.05 versus “aav-shC”/“Cas9-C” groups. Scale bar=100 µm (**J**).

As demonstrated, intratumoral injection of TIMM44-shRNA AAV robustly hindered P1 glioma growth in nude mice, and volumes of aav-shTIMM44 P1 glioma xenografts were significantly lower than the aav-shC xenografts (Figure 7A). A described formula 37 was utilized to calculate the estimated daily tumor growth (in mm^3^ per day), and results demonstrated that aav-shTIMM44 potently inhibited P1 glioma xenograft growth (Figure 7B). At Day-35, all subcutaneous glioma xenografts of the two groups were isolated and weighted (Figure 7C). As shown aav-shTIMM44-injected tumors were much lighter than aav-shC tumors (Figure 7C). There was however no significant difference in the mice body weights between the two groups (Figure 7D). These results showed that intratumoral injection of TIMM44-shRNA AAV potently inhibited P1 glioma xenograft growth in nude mice.

To examine signaling changes *in vivo*, at Day-7 and Day-14 we separated one glioma xenograft per group. Total four xenografts were obtained. Each xenograft was cut into five pieces and were analyzed separately. As shown, *TIMM44* mRNA and protein expression was robustly decreased in aav-shTIMM44-injected xenograft tumors (Figure 7E and F), whereas *TIMM23* mRNA and protein expression was unchanged (Figure 7E and F).

In TIMM44-silenced P1 glioma xenograft tissues, ATP contents were significantly decreased (Figure 7G). Whereas the TBAR activity (Figure 7H) was increased, indicating lipid peroxidation was increased after TIMM44 silencing *in vivo*. Moreover, the relative Caspase-3 activity (Figure 7I), TUNEL-positive staining and the cleaved-PARP level (Figure 7J) were all significantly increased in aav-shTIMM44 xenograft tissues, supporting apoptosis induction. Therefore, in line with the *in vitro* findings, ATP reduction, oxidative injury and apoptosis were detected in TIMM44-silenced subcutaneous P1 glioma xenografts.

Alternatively, using the described protocol 13, 15, 24, P1 glioma cells with the CRISPR/Cas9-TIMM44-KO construct (“koTIMM44”) or the control construct (“Cas9-C”) were intracranially injected into the mouse brains. The patient-derived glioma xenografts (PDX) were established. Twenty-five days later (“Day-25”), the first mouse in the Cas9-C group exhibited obvious symptoms, and all mice were sacrificed and PDX tumors were isolated 15. As shown, compared to the Cas9-C intracranial P1 gliomas, the volumes of koTIMM44 tumors were significantly lower (Figure 7K). The mice body weights were indifferent (Figure 7L). Analyzing tumor tissue lysates, we found that *TIMM44* mRNA and protein expression in intracranial koTIMM44 tumors was depleted (Figure 7M). TIMM23 expression was unaffected (Figure 7M). ATP levels were decreased in koTIMM44 tumors (Figure 7N), and the TBAR intensity was increased (Figure 7O). Thus, TIMM44 KO largely inhibited intracranial P1 glioma xenograft growth in mouse brain.

## Discussion

Glioblastoma and other high grade gliomas have one of the worst prognosis among all malignancies 11, 38, 39. The current clinical treatments, including tumor resection, radiation and temozolomide-based chemotherapies, are not able to significantly improve the overall survival and prognosis for the patients 40-42. It is therefore extremely important to identify novel biomarkers and therapeutic targets of glioma. Increased mitochondrial biogenesis, energy metabolism and oxidative phosphorylation are vital for the tumorigenesis and progression of human glioma 16-18.

Glioma, like other malignant tumors, has aberrant energy metabolism and favors aerobic glycolysis to generate ATP 18. Dysfunctional mitochondria are often observed in glioma 18. Moreover, mitochondrial ultrastructure and proteomics are also dysregulated in glioma 18. These mitochondrial changes play an important role in tumorigenesis and progression of glioma. However, the underlying mechanisms are poorly understood 18. Sun *et al.,* discovered that transferring normal mitochondria of human astrocytes, through endocytosis, could rescue aerobic respiration and enhance radio-sensitivity into glioma cells, and inhibit glioma xenograft growth in nude mice 43.

TIMM44 is a mitochondrial inner membrane translocase protein essential for the import of the nuclear-encoded proteins to the mitochondria 25, 26, 44. Mitochondrial TIMM44 dysfunction, due to genetic variants, could be associated with thyroid carcinoma development 26. Huang *et al.,* has identified TIMM44 as a potential oncogenic gene in colorectal cancer 45. IR-58, a mitochondria-targeting near-infrared autophagy-enhancer, inhibited TIMM44-SOD2 pathway, causing ROS production, oxidative injury, and colorectal cancer cell apoptosis 45.

The results of the present study supported that the mitochondrial protein TIMM44 is an important cancer-promoting molecular for glioma progression. Bioinformatics results and local human glioma tissue data confirmed that *TIMM44* mRNA and protein expression is upregulated in glioma. TIMM44 overexpression in glioma correlated with poor overall survival of the patients. In patient-derived primary glioma cells and immortalized cell lines, TIMM44 shRNA or KO (by CRISPR/Cas9 method) potently inhibited cell viability, proliferation and migration. Conversely, ectopic overexpression of TIMM44 by a lentiviral construct augmented cell proliferation and migration. *In vivo*, the growth of subcutaneous P1 glioma xenografts was suppressed after intratumoral injection of TIMM44 shRNA AAV. Moreover, the growth of intracranial glioma xenografts in nude mice was largely inhibited after TIMM44 KO. These results implied that overexpressed TIMM44 could be a promising and valuable therapeutic target of glioma.

MB-10, through binding to C-terminal region of TIMM44, inhibited mitochondrial protein input, causing viability reduction and apoptosis in cervical cancer HeLa cells 33. Polson *et al.,* showed that KHS101, a synthetic small-molecule, induced glioma cell death by disrupting the mitochondrial chaperone heat shock protein family D member 1 (HSPD1) 46. KHS101 disrupted mitochondrial bioenergetic capacity and glycolytic activity, causing energy crisis in glioma cells 46. Cannabidiol inhibited mitochondrial channel VDAC1 and induced swollen mitochondria, causing apoptosis in glioma cells 47.

We found that overexpressed TIMM44 is essential for maintaining mitochondrial functions in glioma cells. TIMM44 silencing/KO largely inhibited mitochondrial protein input and mitochondrial complex I, causing ATP depletion, mitochondrial membrane potential reduction, oxidative stress and DNA damage, and eventually provoked apoptosis. ATP supplement and the antioxidant NAC can inhibit TIMM44-silencing-induced anti-glioma cell activity. ATP reduction, lipid peroxidation and apoptosis activation were detected as well in TIMM44-depleted subcutaneous and intracranial glioma xenografts. Conversely, TIMM44 overexpression increased cellular ATP contents and alleviated oxidative injury in primary glioma cells. Thus, TIMM44-silencing-induced anti-glioma cell activity was possibly through disrupting mitochondrial functions.

Our previous study has identified an important cancer-promoting function of the mitochondrial protein YME1L in human glioma. YME1L is extremely important for maintaining mitochondrial integrity, morphology, function and plasticity 21, 48, 49. We previously found that YME1L expression is elevated in human glioma tissues and various glioma cells. Our preliminary results found that depletion of YME1L-targeting microRNAs (miRNAs) could be the primary cause of YME1L elevation in human glioma. YME1L depletion, by shRNA or CRISPR/Cas9 KO, potently inhibited glioma proliferation and migration, and induced mitochondrial dysfunctions and apoptosis. On the other hand, overexpression of YME1L augmented glioma cell proliferation and migration. The orthotopic growth of primary glioma xenografts in nude mice was largely inhibited by YME1L depletion.

In the present study, we found that YME1L could be an important upstream protein for TIMM44 in human glioma cells. TCGA database found that *TIMM44* is one of the *YME1L's* top DEGs. TIMM44 expression in primary human glioma cells was decreased following YME1L silencing or KO, but was elevated after ectopic YME1L overexpression. Significantly, YME1L shRNA-induced anti-glioma cell activity was ameliorated after restoring TIMM44 expression using the TIMM44-expressing construct.

Moreover, we found that YEM1L-promoted TIMM44 expression was possibly through promoting GATA3-dependent *TIMM44* transcription. GATA3-*TIMM44* promoter binding in glioma cells was robustly decreased after YME1L silencing or depletion, but was increased after YME1L overexpression. GATA3 silencing significantly decreased *TIMM44* expression. Moreover, GATA3 binding to the *TIMM44* promoter was significantly increased in both human glioma tissues and difference glioma cells. Importantly, YME1L shRNA-induced decrease of GATA3-*TIMM44* promoter binding and TIMM44 downregulation were largely attenuated by exogenously adding ATP in P1 glioma cells. These results supported that *TIMM44* downregulation in YME1L deficient glioma cells could be caused by shutdown of GATA3-dependent *TIMM44* transcriptional machinery (possibly due to mitochondrial stress after YME1L depletion). Upregulation of TIMM44 in glioma could be due to increased *TIMM44* transcriptional machinery through GATA3.

## Conclusion

Overexpressed TIMM44 could be a novel and promising therapeutic target of human glioma.

## Materials and methods

### Reagents, chemicals and antibodies

Polybrene, the antioxidant n-acetylcysteine (NAC), ATP, antibiotics, puromycin and medium were provided by Sigma-Aldrich (St. Louis, MO). The antibodies in this study were from Cell Signaling Technology (Beverly, MA) and Abcam (Cambridge, UK). Fluorescence probes, including TUNEL (terminal deoxynucleotidyl transferase dUTP nick end labeling), MitoTracker Red/MitoTracker Green, tetraethylbenzimidazolylcarbocyanine iodide (JC-1), DAPI (4',6-diamidino-2-phenylindole), EdU (5-ethynyl-20-deoxyuridine), CellROX, dichlorodihydrofluorescein diacetate (DCF-DA), were purchased from Thermo-Fisher Invitrogen (Shanghai, China). MB-10 (“MitoBloCK-10”) 33 was synthesized and verified by Shanghai RuiLu Biotech (Shanghai, China).

### Human tissues and cells

The high grade glioma (HGG, grade III-IV) tissues and matched adjacent brain tissues, derived from sixteen (16) written-informed consent primary patients were reported in our previous studies 13, 15. Fresh tissue lysates were tested by Western blotting and qRT-PCR assays 13, 15. For the immunofluorescence studies, human glioma and xenograft glioma tissue sections (4 µm in thickness) were incubated with anti-TIMM44 antibody overnight and the green fluorescence secondary antibody (for 1h). The mitochondria were co-stained with the mitochondrial marker MitoTracker Red (Invitrogen) and the nuclear marker DAPI (Invitrogen), the fluorescence signals were observed under confocal fluorescence microscope (Zeiss). The primary human glioma cells derived from three written informed-consent glioma patients (“P1”, “P2” and “P3”, as reported early 13, 15, 24), the primary human astrocytes (“Astrocytes1/2”, derived from two patients P1 and P2), immortalized glioma cell lines (A172 and U251MG) were reported in our previous studies 13-15, 50. The protocols of using primary human tissues and cells were according to Declaration of Helsinki, with approval from the Ethics Board of Soochow University.

### shRNA

Two TIMM44 shRNA sequences, two different GATA3 shRNA sequences, and one YME1L shRNA sequence were verified and synthesized by Genechem (Shanghai, China), and were individually sub-cloned into a GV369 construct (Genepharm). The construct along with the lentivirus package constructs (Genechem) were co-transfected to HEK-293 cells, thereby generating shRNA lentivirus, which was filtered, enriched (at MOI=15) and added to glioma cells or astrocytes. For *in vivo* studies, TIMM44 shRNA (-seq1) or the scramble control shRNA was cloned into an adeno-associated virus (AAV) construct (AAV9, Genechem). The shRNA AAV was then generated.

### CRISPR/Cas9

The sequence encoding small-guide (sgRNA) targeting human *TIMM44* (Target DNA Sequence: *CATCTGATAGGTCGACCCAT*, PAM Sequence: *GGG*) or YEM1L 24 was individually inserted into the lenti-CRISPR/Cas9-KO-puro construct (Genechem). The KO construct was then transduced to HEK-293 cells to generate lentivirus. The primary human glioma cells were seeded into six-well plates at 50-60% confluence and transfected with the Cas9-expressing construct (Genechem, Shanghai, China). Stable Cas9-expressing cells were then established after puromycin selection. Cells were then transfected with the lenti-CRISPR/Cas-9 TIMM44 KO construct lentivirus, and puromycin-containing medium was added to select stable cells. TIMM44 KO screening was thereafter performed and the single stable TIMM44 KO cells were established.

### TIMM44/YME1L overexpression

The recombinant GV369 lentiviral construct containing the full-length TIMM44 cDNA (NM_006351.4) or YMEL1 cDNA (NM_139312.2) was transfected to cultured glioma cells. The transfected glioma cells were cultured in puromycin-containing medium and thereafter stable cells were formed. The qRT-PCR and Western blotting assays were carried out to verify TIMM44 overexpression. The control glioma cells were transfected with the empty vector.

### Cellular fluorescence dye assays

As described 51, cells were cultivated at 50-60% confluence and were stained with the designated fluorescence dyes (including JC-1, DCF-DA, CellROX, TUNEL, EdU and DAPI) and further cultured for applied time periods. Fluorescence images were captured under a fluorescence microscopy (Leica). It intensity was recorded from five random views of each treatment.

### Cell migration and invasion

The described cells (at 5 × 10^4^ cells per chamber, in serum-free medium) were initially seeded onto the upper surface of the “Transwell” chamber (BD Biosciences). To the lower compartment, the complete medium with serum was added. After 24h, the migrated cells passing through were fixed and stained 51. For *in vitro* cell invasion assays, the chamber surface was coated with Matrigel (Sigma), and other steps were the same.

### TBAR assay

Lipid peroxidation in cell and tissue lysates (30 µg per sample) was tested by a thiobarbituric acid reactive substance (TBAR) kit (Cayman Chemical, MI) based on the attached protocol. The kit reagent colorimetrically quantified lipid peroxidation and measured malondialdehyde (MDA). The optical density of TBAR was tested at 545 nm with the reference wavelength at 600 nm at room temperature. It was expressed in nmol per mg of total protein and was always normalized to that of control.

### Mitochondrial protein import assays

The detailed protocols were described elsewhere 33. In brief, prior to import into purified mitochondria, [^35^S]-methionine and cysteine labeled Su9-DHFR (dihydrofolate reductase) fusion protein (based on the described protocol 33, 52, 53, provided by Shanghai Genechem Co.) was generated with the TNT Quick Coupled Transcription/Translation kit (Promega, Shanghai, China). The isolated mitochondria (200 µg/mL) of human glioma cells was dissolved to the described import buffer 33 and import reaction was started by the addition of translation mix. After 15 min, the import was terminated by trypsin on ice and soybean trypsin inhibitor was thereafter added. Mitochondria were pelleted by centrifugation, disrupted in Laemmli sample buffer and analyzed by SDS-PAGE and autoradiography.

### Other assays

The quantitative real-time PCR (qRT-PCR), Western blotting, cell counting kit-8 (CCK-8), Trypan blue staining of cell death, the Caspase-3 activity assay, single strand DNA (ssDNA) ELISA were described in our previous studies 13-15, 50, 54, 55. ATP contents in cellular and tissue lysates were measured as previously described 56. The mitochondrial complex I activity in glioma cells with applied treatments was analyzed by a commercial kit (Abcam) according to the attached protocols.

### Chromatin immunoprecipitation (ChIP) assay

As described 55 cell lysates were treated by a MisonixSonicator 3000 Homogenizer 57 to achieve fragmented genomic DNA. Lysates were diluted with ChIP dilution buffer and were immunoprecipitated with the anti-GATA3 (ab199428, Abcam) antibody. GATA3-bound DNA was eluted from the protein A/G agarose, and NaCl was included. DNA containing the proposed conserved *TIMM44* promoter site (GAGATAGGG) was analyzed via quantitative PCR (qPCR).

### Xenograft studies

The athymic nude mice, half male, half female, 6-7 week old, 18.2-19.5 g weight, were purchased from the Shanghai Laboratory Animal Center (SLAC, Shanghai, China) and were maintained under standard conditions. The primary human glioma cells P1 were trypsinized, washed and re-suspended. Cells (in 200 µL of Matrigel basic medium) were subcutaneously injected into the right flanks of the mice. Within three weeks of cell inoculation, the P1 glioma xenografts were established and tumor volume close to 100 mm^3^. The mice were intratumorally injected with adeno-associated virus (aav)-packed shRNA (1×10^9^ PFU, 10 µL). The mice body weights and the tumor volumes (15) were recorded every six days. For intracranial glioma xenograft experiments, P1 glioma cells were intracranially injected as described 58. Magnetic resonance imaging (MRI) was utilized to visualize the intracranial xenografts. On Day-25, all mice were sacrificed and xenografts were isolated. Tumor volumes were calculated by the described formula 15. The animal studies were approved by Institutional Animal Care and Use Committee and Ethics Committee of Soochow University.

### Statistical analyses

Data in this study were always with normal distribution and were expressed as means ± standard deviation (SD). To examine statistical differences between three and more groups, one-way ANOVA and a Scheffe's f-test (SPSS 23.0, SPSS Co., Chicago, CA) were utilized. The two-tailed unpaired t test (Excel 2007) was utilized to examine significance between two groups. *P* values < 0.05 were considered statistically significant.

## Supplementary Material

Supplementary figures.

## Abbreviations

AAV: Adeno-associated virus
CNS: central nerve system
DAPI: 4',6-diamidino-2-phenylindole
EdU: 5-ethynyl-20-deoxyuridine
CPT1A: carnitine palmitoyltransferase 1A
DHFR: dihydrofolate reductase
DCF-DA: dichlorodihydrofluorescein diacetate
GTEx: Genotype-Tissue Expression
GBM: glioblastoma
KLF4: Krüppel-like factor 4
IHC: immunohistochemistry
KO: knockout
Mfn1: mitofusin-1
Mfn2: mitofusin-2
Opa1: optic atrophy 1
ROS: reactive oxygen species
ROC: receiver operating characteristic
SD: standard deviation
TIMM44: translocase of inner mitochondrial membrane 44
ssDNA: single strand DNA
YME1L: YME1 Like 1 ATPase
TUNEL: terminal deoxynucleotidyl transferase dUTP nick end labeling
JC-1: tetraethylbenzimidazolylcarbocyanine iodide
TBAR: thiobarbituric acid reactive substance
