# Supplementary material for: TIMM44 is a potential therapeutic target of human glioma
Source: Theranostics. 2022 Oct 31;12(17):7586–602. doi: 10.7150/thno.78616 (PMC9691352; doi:10.7150/thno.78616)
Supplement: Supplementary file 1 — Supplementary figures. [file thnov12p7586s1.pdf]

Figure S1

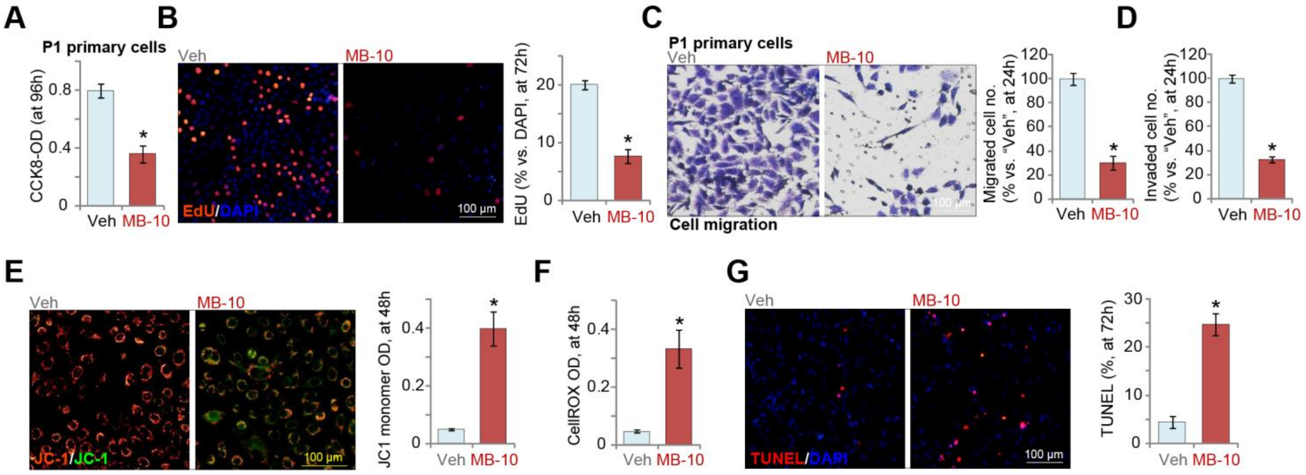

Figure S1. P1 glioma cells were treated with MitoBloCK-10 (MB-10, for 25 μ M) or the vehicle control ("Veh", 0.1% DMSO), and cells were further cultivated for designated hours; Cell viability, proliferation, migration and invasion were examined by CCK-8 (**A**), nuclear EdU staining (**B**), Transwell (**C**) and "Matrigel Transwell" (**D**) assays, respectively. Mitochondrial depolarization (by measuring JC-1 green monomers, **E**), CellROX intensity OD (**F**) and cell apoptosis (TUNEL assays, **G**) were tested as well. The data were presented as mean ± standard deviation (SD). * ***P*** < 0.05 *vs.* "Veh". Experiments were repeated five times with similar results obtained. Scale bar = 100 μm.

Figure S2

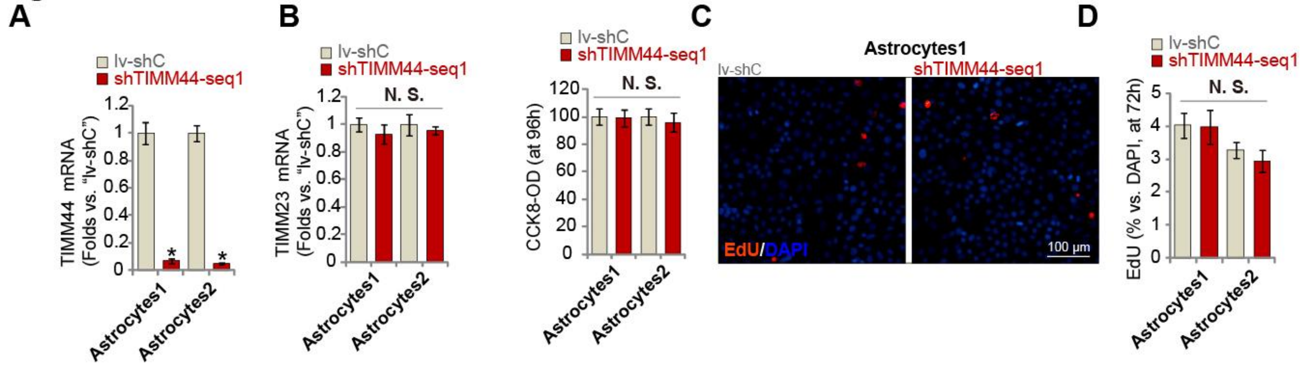

Figure S2. Expression of listed mRNAs in the primary human astrocytes ("Astrocytes1/2", derived from two patients) with the TIMM44 shRNA ("shTIMM44-seq1") or the lentiviral scramble control shRNA ("lv-shC") was shown (**A** and **B**). Astrocytes were further cultivated for designated hours, and cell viability (**C**) and proliferation (**D**) were tested. The data were presented as mean ± standard deviation (SD). * ***P*** < 0.05 *vs.* "lv-shC". "N.S." stands for non-statistical difference (***P*** > 0.05). The *in vitro* experiments were repeated five times with similar results obtained. Scale bar = 100 μm.

Figure S3

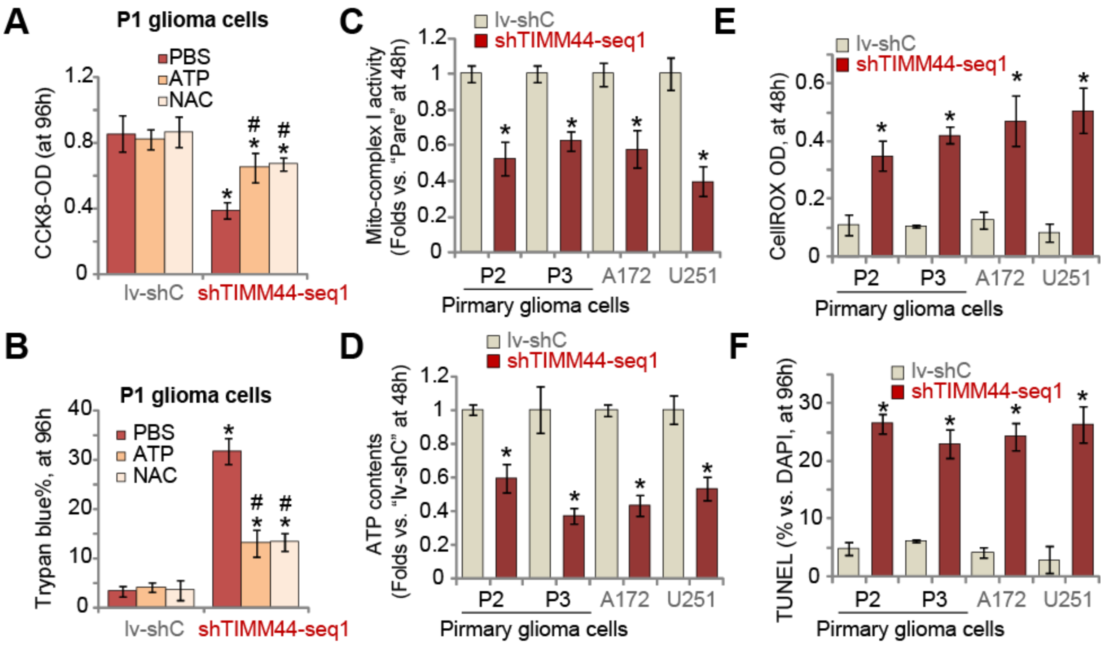

Figure S3. P1 glioma cells with the TIMM44 shRNA (shTIMM44-seq1) or the lentiviral scramble control shRNA ("lv-shC") were treated with ATP (2 mM), N-Acetyl-L-cysteine (NAC, 400 μ M) or PBS for 96h, cell viability (**A**) and death (**B**) were examined by CCK-8 and Trypan blue staining assays, respectively. The primary glioma cells ("P2" and "P3") or established lines (A172 and U251) with the TIMM44 shRNA (shTIMM44-seq1) or the lentiviral scramble control shRNA ("lv-shC") were cultivated for designated hours, the relative mitochondrial complex I activity (**C**), cellular ATP contents (**D**), CellROX intensity OD (**E**) and nuclear TUNEL ratio (**F**) were tested. The data were presented as mean ± standard deviation (SD). * $\boldsymbol{P} < 0.05$ *vs.* "lv-shC". # $\boldsymbol{P} < 0.05$. "PBS" (**A** and **B**). The *in vitro* experiments were repeated five times with similar results obtained.

Figure S4

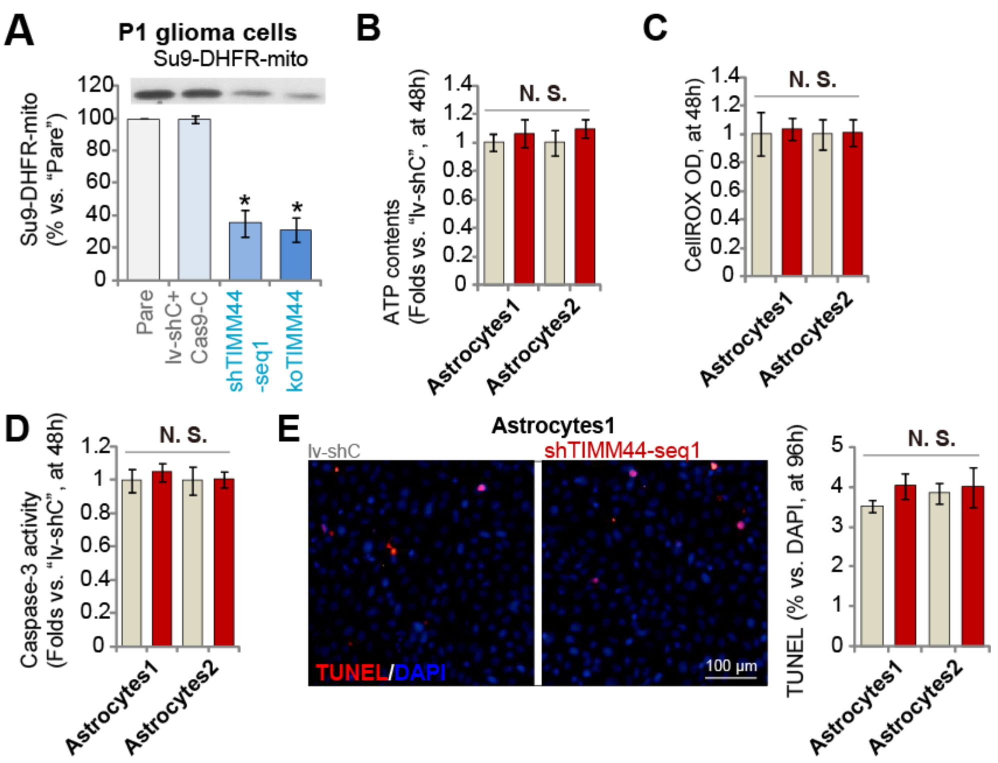

Figure S4. The isolated mitochondria (200 μg/mL) of P1 glioma cells with applied genetic modifications was incubated with [^{35}S]-methionine and cysteine-labeled precursor Su9-DHFR protein, and mitochondria protein import was stopped by adding trypsin after 15 min. The mitochondrial Su9-DHFR protein ("Su9-DHFR-mito") was tested by SDS-PAGE and autoradiography (**A**), the relative level of Su9-DHFR-mito was quantified from five repeats (**A**). The primary human astrocytes ("Astrocytes1/2", derived from two patients) with the TIMM44 shRNA ("shTIMM44-seq1") or the lentiviral scramble control shRNA ("lv-shC") were cultivated for designated hours, cellular ATP contents (**B**), CellROX intensity OD (**C**), Caspase-3 activity (**D**) and cell apoptosis (**E**) were tested. The data were presented as mean ± standard deviation (SD). * $P < 0.05$ *vs.* "Pare". "N. S." stands for non-statistical difference ($P >$ 0.05). The *in vitro* experiments were repeated five times with similar results obtained. Scale bar = 100 μm.

Figure S5

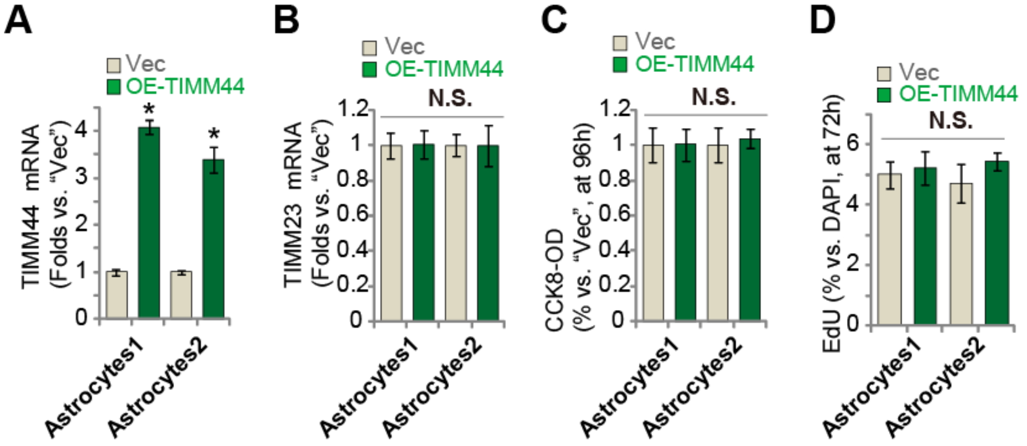

Figure S5. Expression of listed mRNAs in the primary human astrocytes ("Astrocytes1/2", derived from two patients) with the lentiviral TIMM44-expressing vector ("OE-TIMM44") or the empty vector ("Vec") was shown (**A** and **B**). Astrocytes were further cultivated for designated hours, and cell viability (**C**) and EdU-positive nuclei ratio (**D**) were tested. The data were presented as mean ± standard deviation (SD). * ***P*** < 0.05 *vs.* "Vec". "N.S." stands for non-statistical difference (***P*** > 0.05). The *in vitro* experiments were repeated five times with similar results obtained.

Figure S6

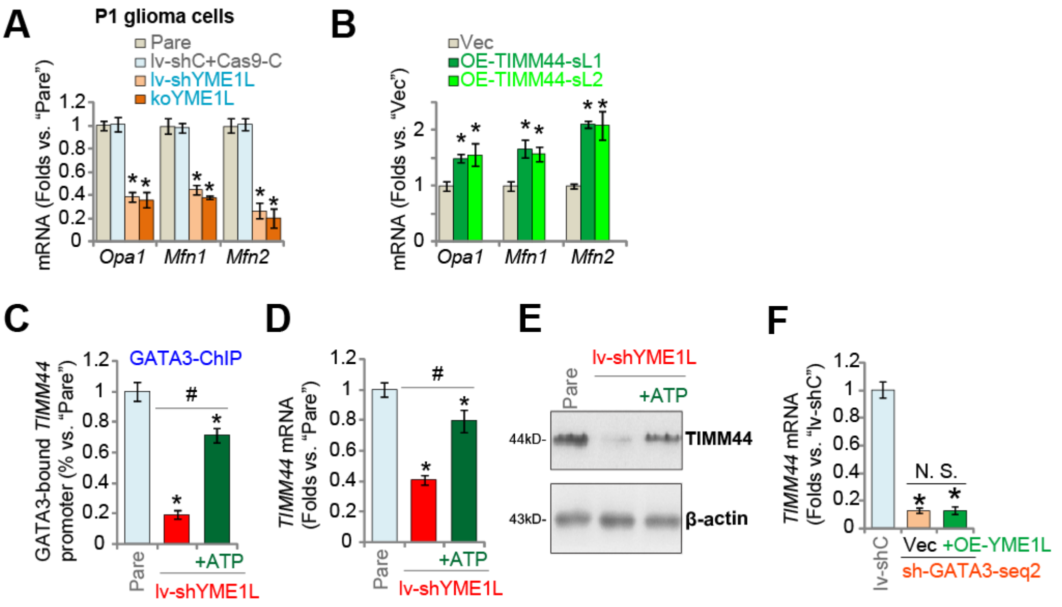

Figure S6. P1 primary human glioma cells, stably expressing the YME1L shRNA (lv-shYME1L), the lenti-CRSIPR/Cas9-YME1L-KO-puro construct ("koYME1L"), the lentiviral scramble shRNA plus the CRSIPR/Cas9 empty vector ("lv-shC+Cas9-C"), the YME1L-expressing construct ("OE-YME1L-sL1 or OE-YME1L-sL2", two stable selections) or the empty vector ("Vec"), were established, expression of listed mRNAs was shown (**A** and **B**). P1 primary human glioma cells, stably expressing the YME1L shRNA (lv-shYME1L) were treated with or without ATP (2 mM) for 24h, chromosome IP (ChIP) showed the relative levels of the proposed *TIMM44* promoter binding to GATA3 protein in the nuclei (**C**); Expression of *TIMM44* mRNA (**D**) and listed proteins (**E**) was tested. The sh-GATA3-seq2-expressing P1 glioma cells were further transduced with or without the lentiviral YME1L-expressing construct ("+OE-YME1L"), stable cells were formed by selection, and expression of *TIMM44* mRNAwas tested (**F**). The data were presented as mean ± standard deviation (SD). * ***P*** < 0.05 *vs.* "Pare"/"Vec"/"lv-shC". # ***P*** < 0.05. "N.S." stands for non-statistical difference (***P*** > 0.05). The experiments were repeated five times with similar results obtained.
